# Activation of BNIP3-mediated mitophagy protects against renal ischemia–reperfusion injury

**DOI:** 10.1038/s41419-019-1899-0

**Published:** 2019-09-12

**Authors:** Chengyuan Tang, Hailong Han, Zhiwen Liu, Yuxue Liu, Lijun Yin, Juan Cai, Liyu He, Yu Liu, Guochun Chen, Zhuohua Zhang, Xiao-Ming Yin, Zheng Dong

**Affiliations:** 1 Department of Nephrology, The Second Xiangya Hospital, Central South University, Hunan Key Laboratory of Kidney Disease and Blood Purification, Changsha, Hunan China; 2 Institute of Molecular Precision Medicine, Xiangya Hospital and Center for Medical Genetics, Central South University, Changsha, Hunan China; 3 Department of Pathology and Laboratory Medicine, Indiana University School of Medicine, Indianapolis, IN USA

**Keywords:** Mitophagy, Acute kidney injury

## Abstract

Acute kidney injury (AKI) is a syndrome of abrupt loss of renal functions. The underlying pathological mechanisms of AKI remain largely unknown. BCL2-interacting protein 3 (BNIP3) has dual functions of regulating cell death and mitophagy, but its pathophysiological role in AKI remains unclear. Here, we demonstrated an increase of BNIP3 expression in cultured renal proximal tubular epithelial cells following oxygen-glucose deprivation-reperfusion (OGD-R) and in renal tubules after renal ischemia–reperfusion (IR)-induced injury in mice. Functionally, silencing *Bnip3* by specific short hairpin RNAs in cultured renal tubular cells reduced OGD-R-induced mitophagy, and potentiated OGD-R-induced cell death. In vivo, *Bnip3* knockout worsened renal IR injury, as manifested by more severe renal dysfunction and tissue injury. We further showed that *Bnip3* knockout reduced mitophagy, which resulted in the accumulation of damaged mitochondria, increased production of reactive oxygen species, and enhanced cell death and inflammatory response in kidneys following renal IR. Taken together, these findings suggest that BNIP3-mediated mitophagy has a critical role in mitochondrial quality control and tubular cell survival during AKI.

## Introduction

Acute kidney injury (AKI) is a clinical syndrome of abrupt loss of renal functions that is pathologically featured by sublethal and lethal injury of renal tubules. The clinical risk factors for AKI mainly include renal ischemia–reperfusion (IR), nephrotoxins, and sepsis. AKI has poor short- and long-term outcome. Besides its high short-term mortality, AKI has been recently recognized as an important risk factor for the development of chronic kidney disease (CKD)^1,2^. Recent experimental studies have demonstrated that the pathogenesis of AKI is complex, involving tubular, microvascular, and inflammatory mechanisms, diverse signal pathways, and molecules^3^. Of note, studies have highlighted a pivotal role of mitochondrial dysfunction in AKI development and progress^4–9^. Targeting mitochondrial protection and timely removal of damaged mitochondria have been proposed as promising strategies to prevent, treat AKI, and impede AKI-CKD transition^10–13^.

Mitophagy is a specific form of autophagy that selectively degrades mitochondria. As a critical component of mitochondrial quality control, mitophagy targets damaged or dysfunctional mitochondria for prompt elimination, thereby preventing excessive reactive oxygen species (ROS) production, and release of mitochondrial pro-apoptotic factors and damage-associated molecular patterns (DAMPs) that may promote pathological inflammatory responses^6,14^. Mechanistically, mitophagy requires a coordinative induction of autophagy and mitochondrial priming for autophagic recognition. Thus, two mitochondrial priming mechanisms have been proposed, which rely on PTEN-induced kinase 1 (PINK1)-parkin RBR E3 ubiquitin protein ligase (PARK2) pathway and mitophagy receptors BCL2-interacting protein 3 like (BNIP3L/NIX), BCL2-interacting protein 3 (BNIP3), or FUN14 domain containing 1^15,16^. Recent evidence from other studies and our study suggests an involvement of mitophagy in AKI pathogenesis^17–21^. However, its precise roles and regulation in AKI remain not fully understood.

BNIP3 is a Bcl-2 family protein with an atypical BH3 domain that primarily localizes in mitochondrial out membrane. BNIP3 was firstly identified as a pro-apoptotic protein. Its induction has been shown to sensitize BAX (BCL2-associated X, apoptosis regulator) and BAK (BCL2 antagonist/killer 1) insertion and activation in the mitochondria, a key step for mitochondrial outer membrane permeabilization that results in the release of pro-apoptotic factors in the inter-membrane mitochondrial space into the cytosol to initiate apoptosis cascade^22,23^. On the other hand, recent studies suggest that BNIP3 is also a mitophagy receptor, and plays pro-survival roles in some pathological conditions^24–27^. In kidney, emerging evidence indicated that BNIP3 might have a role in the regulation of mitophagy in cultured renal proximal tubular cells (RPTCs) in response to oxidative stress and hypoxia^20^, but its precise role in AKI pathogenesis remains largely unknown. In the present study, we have investigated the role of BNIP3 in the development of ischemic AKI by using cultured RPTCs subjected to oxygen-glucose deprivation and reoxygenation (OGD-R) and a mouse model of renal IR injury as model systems. The results support that BNIP3 is an important mediator of mitophagy in renal tubular cells, and BNIP3-dependent mitophagy plays a critical role in mitochondrial quality control for tubular cell viability and function in AKI.

## Results

### Suppression of *Bnip3* expression sensitizes BUPMT cells to OGD-R injury in vitro

**Fig. 1:**
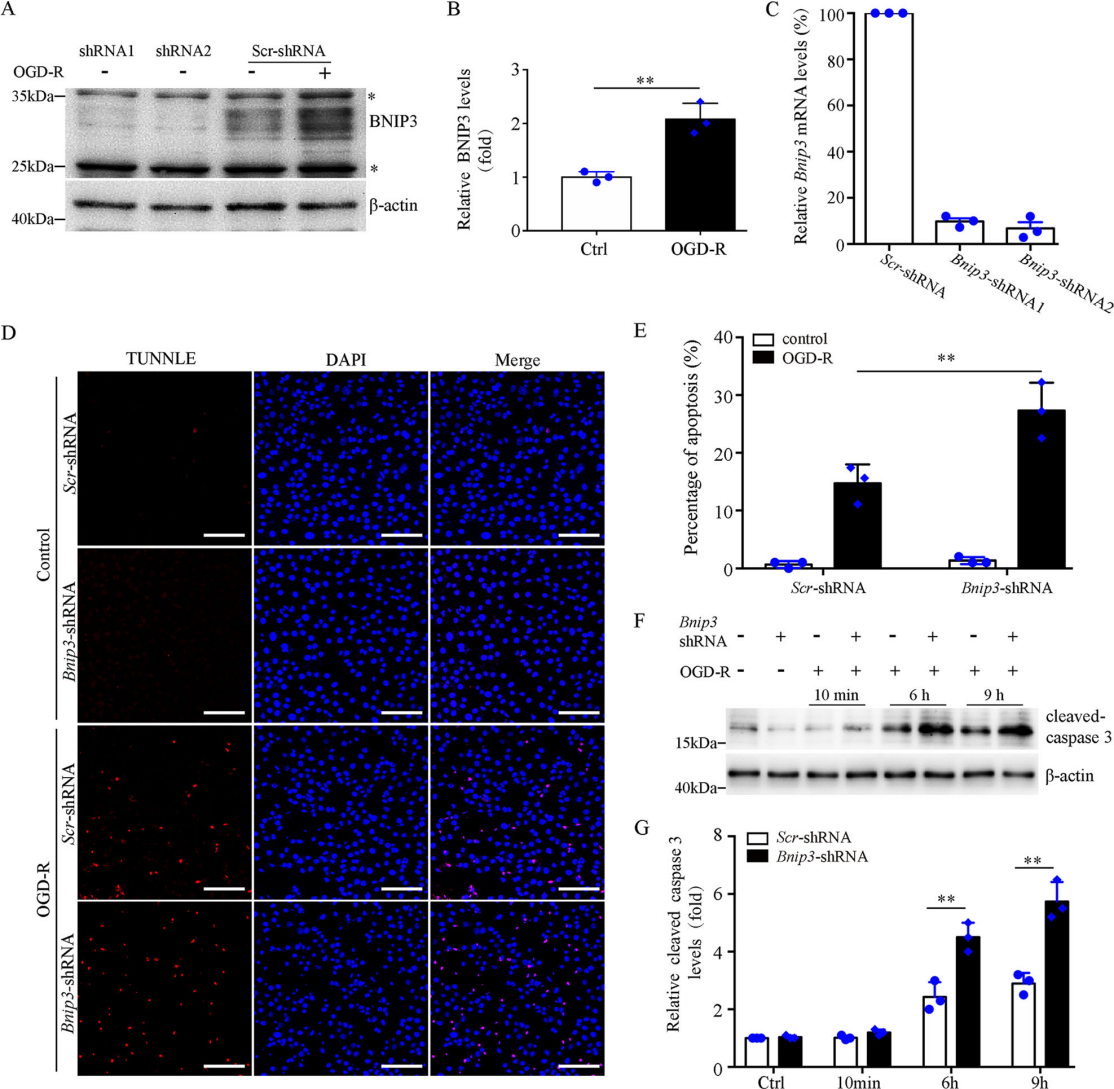
Suppression of *Bnip3* expression sensitizes BUMPT cells to OGD-R injury. **a** Representative immunoblot of BNIP3. For OGD-R treatment, cells were subjected to 2 h OGD followed by 6 h reperfusion. Note: * indicated unspecific band. **b** Densitometry of BNIP3 signals in BUMPT cells with or without OGD-R treatment (*n* = 3). The BNIP3 signals were normalized to the β-actin signal of the same samples to determine the ratios. **c** Related *Bnip3* mRNA levels in BUMPT cells stably expressing scrambled (Scr) shRNA or *Bnip3-*shRNA (*n* = 3). The *Bnip3* mRNA levels were normalized to the *Gaphd* mRNA levels of the same sample to determine the rations. The ratios of control cells (Ctrl) were arbitrarily set as 1. **d** Representative images of TUNEL assay. Bar: 100 μm. **e** Apoptosis percentage (*n* = 3). **f** Representative immunoblots of active caspase-3. Cells were subjected to 2 h OGD followed by reperfusion for 10 min, 6 h, or 9 h, and whole-cell lysates were collected for immunoblot of activated caspase-3 and β-actin. **d** Densitometry of active caspase-3 (*n* = 3). The signal of cleaved caspase-3 was normalized to the β-actin signal of the same samples to determine the ratios. The ratios of control cells (Ctrl) were arbitrarily set as 1. Each symbol (circle and diamond) represents an independent experiment. Error bars: SEM. **p < 0.01

We first evaluated the expression of BNIP3 in Boston University mouse proximal tubule (BUMPT) cells that were subjected to OGD-R to mimic in vivo IR. Immunoblotting analysis showed a dramatic increase of BNIP3 in BUMPT cells after OGD-R (Fig. 1b, c). Specific *Bnip3-*short hairpin RNAs (shRNAs) dramatically reduced BNIP3 expression (Fig. 1a, c). Terminal deoxynucleotidyl transferase-mediated dUTP nick-end labeling (TUNEL) assay demonstrated that *Bnip3* knockdown (KD) showed minimal effect on TUNEL labeling under controlled condition, but dramatically increased the number of TUNEL-positive cells after OGD-R (Fig. 1d, e). Consistently, immunoblot of active/cleaved caspase-3 demonstrated that *Bnip3* KD cells had a significantly higher level of activated caspase-3 than wild-type (WT) cells after OGD-R (Fig. 1f, g). Taken together, these findings support that *BNIP3* silencing increases the sensitivity of proximal tubular cells to OGD-R-induced apoptosis, suggesting a pro-survival role of BNIP3 in these cells.

### Suppression of *Bnip3* expression reduces OGD-R-induced mitophagy in BUPMT cells

**Fig. 2:**
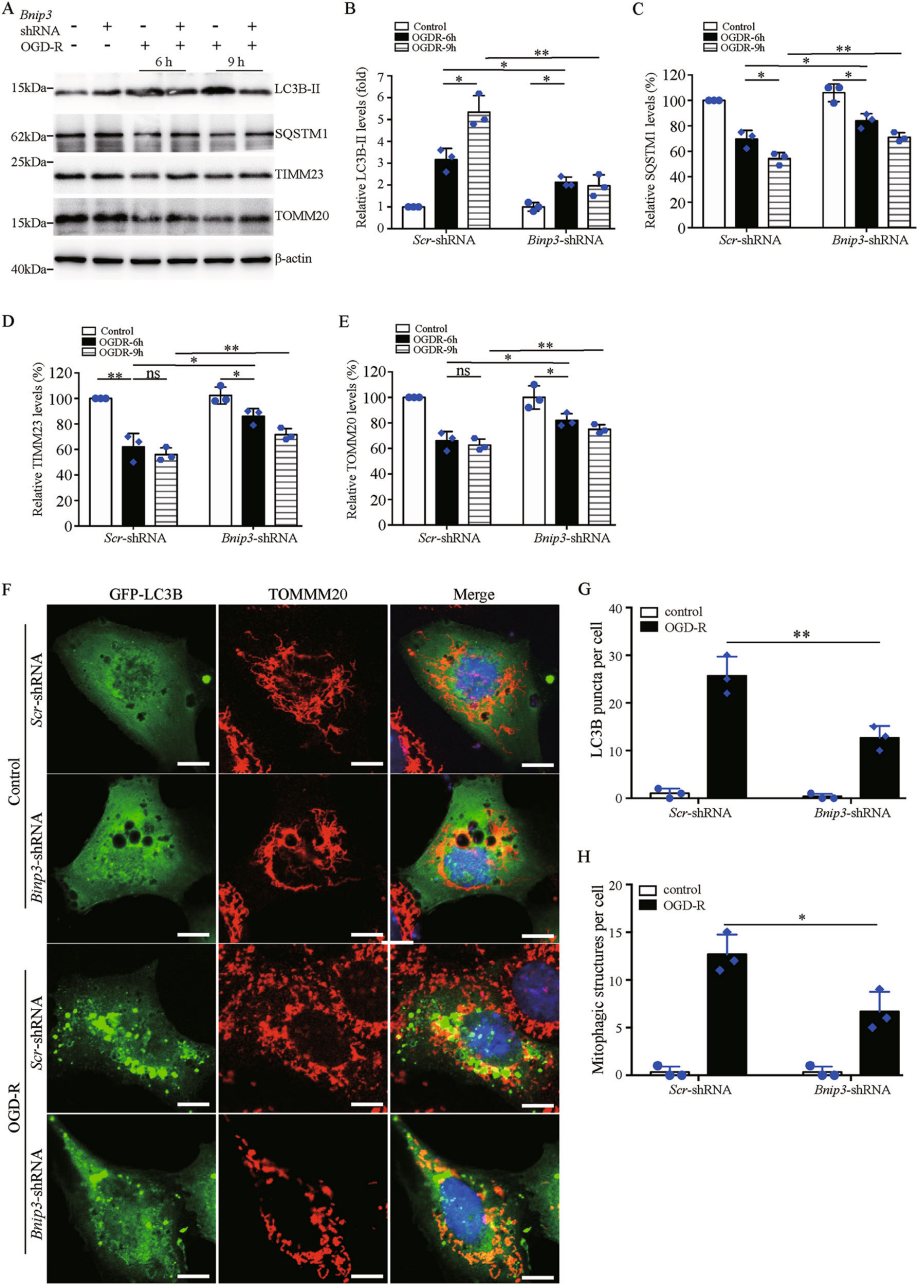
Suppression of *Bnip3* expression reduces OGD-R-induced mitophagy in BUMPT cells. **a** Representative blots. BUMPT cells stably expressing *Scr*-shRNA or *Bnip3*-shRNA were subjected to 2 h of OGD, followed by reperfusion for 6 or 9 h. Whole-cell lysates were collected for immunoblot of LC3B-II, SQSTM1, TIMM23, TOMM20, and β-actin. **b**–**e** Densitometry of LC3B-II (**b**), SQSTM1 (**c**), TIMM23 (**d**), and TMOM20 (**e**) signals (*n* = 3). For densitometry, the signals of the target proteins were normalized to the β-actin signal of the same samples to determine the ratios. The ratios in sample of *Scr*-shRNA cells without OGD-R were arbitrarily set as 1 or 100% in each blot. **f** Representative images of autophagosome and mitophagosome. Bar: 10 μm. BUMPT cells stably expressing *Scr*-shRNA or *Bnip3*-shRNA were transiently transfected with GFP-LC3B to label autophagosome. At 24 h after transfection, cells were subjected to 2 h of OGD, followed by 6 h of reperfusion. Cells cultured in regular medium and incubator were used as control. The cells were then fixed and stained with primary antibody against TOMM20 and corresponding fluorescent-labeled secondary antibody to label mitochondria. Nuclei were stained with DAPI (blue). **g** Quantification for LC3B puncta. The number of LC3B puncta in at least 30 cells from three different experiments was counted to indicate autophagosome formation. **h** Quantification for mitophagosome formation. LC3B puncta colocalizing with mitochondria in at least 30 cells from three different experiments were counted to indicate mitophagosome formation. Each symbol (circle and diamond) represents an independent experiment. Error bars: SEM, *n* = 3. *P < 0.05; **p < 0.01; ns not significant

BNIP3 regulates both cell death and mitophagy. Our above results showed pro-survival functions of BNIP3 in BUPMT cells (Fig. 1d–g). We therefore focused on its potential role in the regulation of mitophagy. Immunoblotting analysis showed a remarkable increase of autophagosome marker microtubule-associated protein 1 light chain 3β (MAP1LC3B/LC3B-II) and a decrease of specific autophagy substrate sequestosome 1 (SQSTM1) in BUPMT cells following OGD-R, indicating autophagy activation (Fig. 2a–c). Moreover, the alterations in LC3B-II and SQSTM1 were associated with a marked reduction of mitochondrial membrane protein translocase of inner mitochondrial membrane 23 (TIMM23) and translocase of outer mitochondrial membrane 20 (TOMM20) (Fig. 2a, d, e), suggesting an induction of mitophagy. Notably, *Bnip3* KD resulted in less LC3B-II accumulation, and partially reduced the degradation of SQSTM1 as well as TIMM23 and TOMM20 in BUMPT cells following OGD-R (Fig. 2a–e). Collectively, these findings suggested an important role of BNIP3 in the regulation of mitophagy in BUPMT cells during OGD-R. To further verify the pro-mitophagy function of BNIP3, we evaluated mitophagosome formation by assessing the colocalization of mitochondria and autophagosomes. As shown in Fig. 2f, under controlled condition, both WT and *Bnip3* KD cells had very few green fluorescent protein (GFP)-LC3B puncta, indicating a low level of autophagy. In the setting of OGD-R, an increase of GFP-LC3B puncta occurred in both WT and *Bnip3* KD cells, and partial GFP-LC3B puncta colocalized with the mitochondria (Fig. 2f), suggesting the formation of mitophagosomes. Notably, quantification analysis showed that OGD-R induced much less autophagosome and mitophagosome formation in *Bnip3*-KD cells than in WT cells, suggesting an inhibitory effect of *Bnip3* KD on mitophagy (Fig. 2g, f). Taken together, these results suggest a pro-mitophagy role of BNIP3 in RPTCs.

### *Bnip3* deficiency exacerbates renal IR-induced kidney injury in vivo

**Fig. 3:**
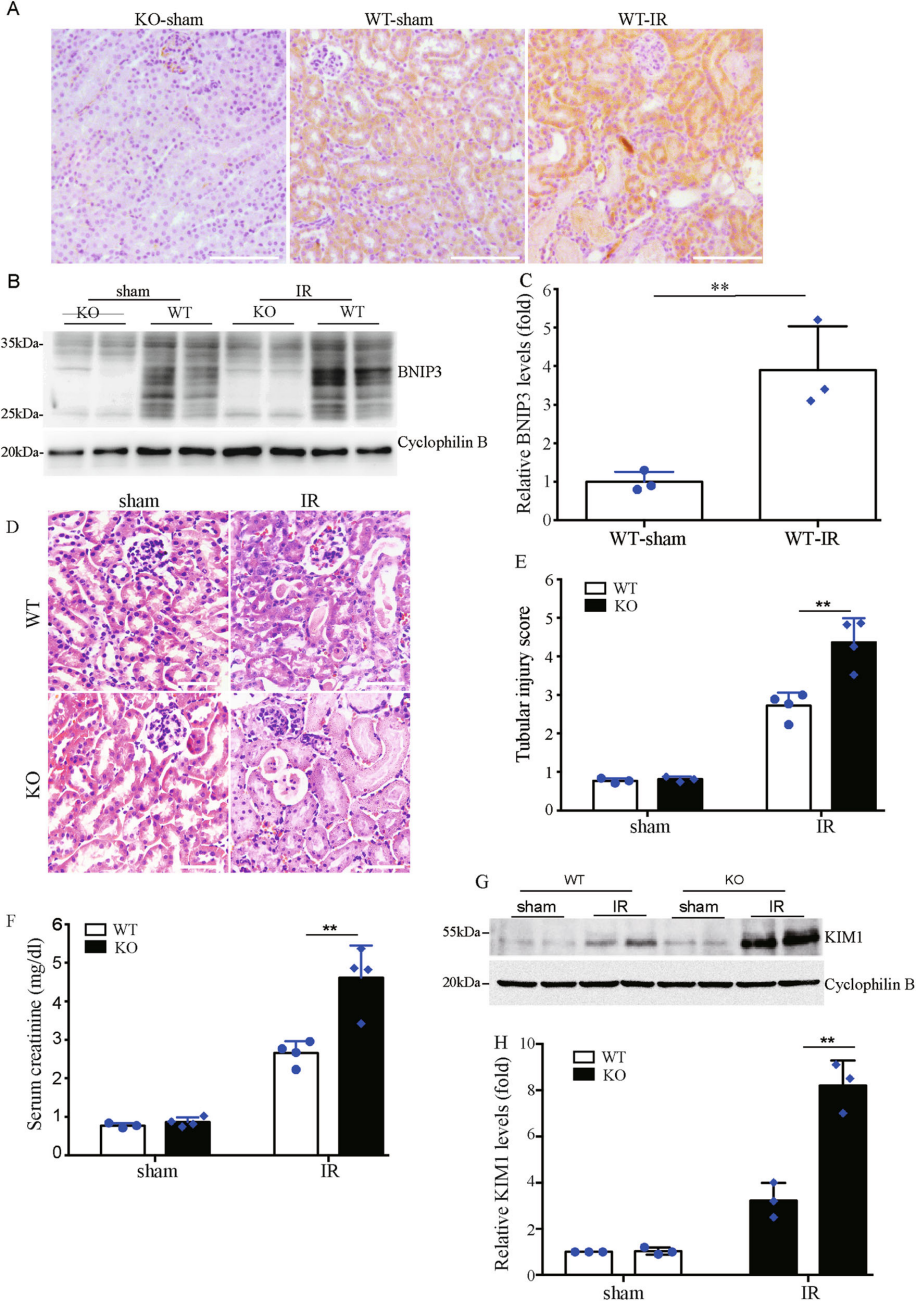
*Bnip3* deficiency exacerbates renal IR-induced kidney injury. *Bnip3* deficiency (KO) mice and their wild-type littermates (WT) (male, 8 weeks old) were subjected to 30 min bilateral renal ischemia followed by 48 h of reperfusion (IR) or sham operation (sham). Kidney tissues were collected for histological and biochemical analysis. **a** Representative images of BNIP3 staining. **b** Representative immunoblot of BNIP3. **c** Densitometry of BNIP3 signals (*n* = 3). For densitometry, the BNIP3 signals were normalized to the cyclophilin B signal of the same samples to determine the ratios. **d** Representative images of hematoxylin–eosin staining. Bar: 100 μm. **e** Pathological score of tubular damage (*n* = 4). **f** Blood samples were collected for measurements of serum creatinine (*n* = 4). **g** Representative immunoblot of KIM1. **h** Densitometry of KIM1 signals (*n* = 4). The KIM1 signals were normalized to the cyclophilin B signal of the same samples to determine the ratios. The ratios of sham-operated WT were arbitrarily set as 1. Each symbol (circle and diamond) represents an individual mouse. Error bars: SEM. **p < 0.01

We then determined the role of BNIP3 in the pathogenesis of ischemic AKI in vivo. We first examined the expression of BNIP3 in kidney tissues of mouse models of ischemic AKI that was induced by 30 min of bilateral kidney ischemia, followed by 48 h of reperfusion. Immunohistochemical analysis showed that BNIP3 was dramatically induced in cortical renal proximal tubules of ischemic mice (Fig. 3a). Immunoblotting analysis confirmed the induction of BNIP3 in kidney tissues following renal IR (Fig. 3b, c). The above finding provided in vivo evidence for the induction of BNIP3 in RPTCs in ischemic AKI. To verify the role of BNIP3 in the pathogenesis of ischemic AKI, *Bnip3*-knockout (KO) mice were applied (Fig. 3a, b). Histological examination by hematoxylin and eosin (HE) staining showed that both *Bnip3*-KO mice and their WT control littermates had normal kidney structure under physiological condition, but showed obvious tubular injury in the setting of renal IR (Fig. 3d). Quantitative analysis showed that KO mice had a significantly higher renal tubular damage score than WT mice after renal IR (Fig. 3e). In line with the results of histological analysis, serum creatinine concentration in both KO and WT mice was low and comparable following sham operation (Fig. 3f), and was dramatically increased in both mice after renal IR. However, the concentration of serum creatinine in ischemic KO was significantly higher than in WT mice in the same experimental setting (Fig. 3f), indicating more severe renal failure in *Bnip3*-KO mice after renal IR. Moreover, renal IR induced significantly higher levels of kidney injury molecule 1 (KIM1), a biomarker of AKI, in *Bnip3-* KO mice than in WT mice (Fig. 3g, h).

**Fig. 4:**
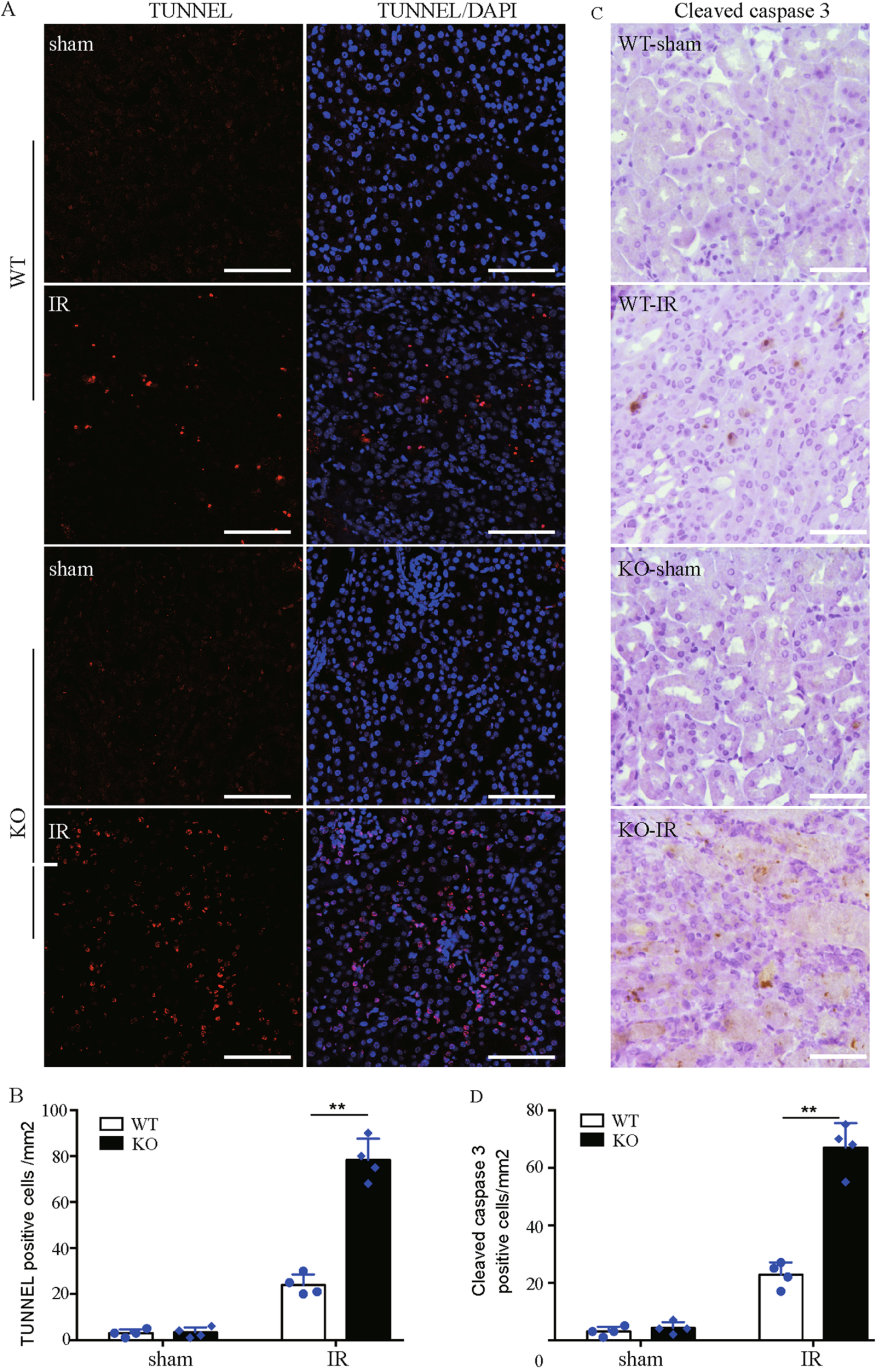
*BNIP3* deficiency increases tubular cell apoptosis after renal IR injury. *BNIP3*-KO and WT mice were subjected to renal IR or sham operation (sham). **a** Representative images of TUNEL staining of kidney tissues. Bar: 75 μm. **b** Quantification of TUNEL-positive cells in kidney tissues, *n* = 3. **c** Representative images of histochemical staining of activated caspase-3 in kidney tissues. Bar: 200 μm. **d** Quantification of activated caspase-3-positive cells in kidney tissues, *n* = 3. Each symbol (circle and diamond) represents an individual mouse. Error bars: SEM. **p < 0.01

The effect of *Bnip3* deficiency on renal tubular cell apoptosis was also evaluated by TUNEL assay and staining of active cleaved caspase-3. As shown in Fig. 4a, *Bnip3*-KO and WT kidneys had few TUNEL-positive tubular cells following sham operation condition, and both had an increase of the positive cells after renal IR. Quantification analysis demonstrated that there were significantly more TUNEL-labeled tubular cells in *Bnip3*-KO kidneys than in WT kidneys after renal IR (Fig. 4b). Consistently, staining and quantification analysis demonstrated that renal IR resulted in significantly more active caspase 3-positive tubular cells in *Bnip3*-KO mice than in WT mice (Fig. 4c, d). Collectively, these in vivo findings provided compelling evidence supporting a renoprotective role of BNIP3 in ischemic AKI.

### *Bnip3* deficiency increases renal inflammation after renal IR

**Fig. 5:**
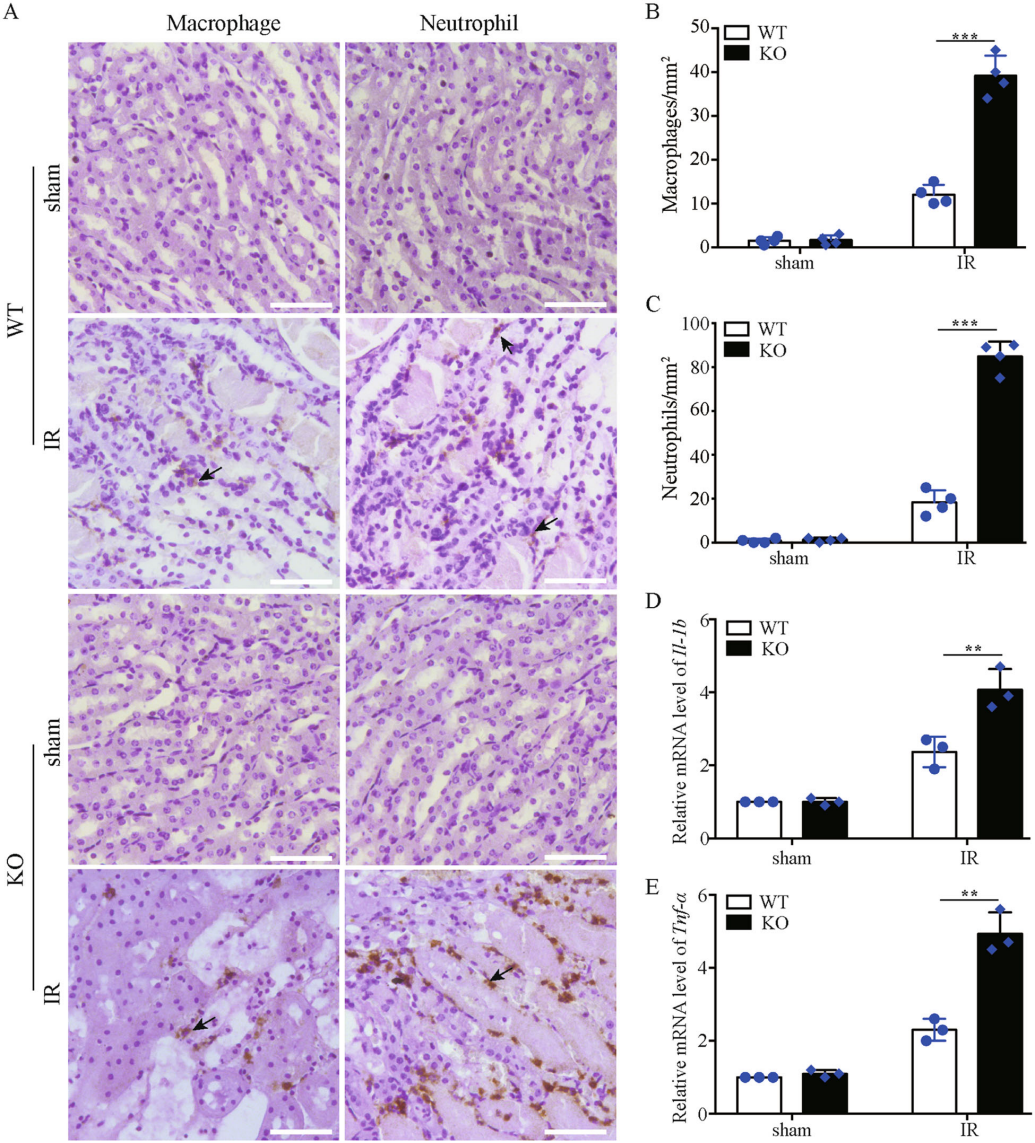
*Bnip3* deficiency enhances inflammation response in kidney following renal IR. *Bnip3*-KO and WT mice were subjected to renal IR or sham operation (sham). Kidney tissues were collected for analysis of renal infiltration of macrophages and neutrophils, and for measurement of the mRNA levels of inflammatory cytokine IL-1b and TNF-α. **a** Representative images of macrophage and neutrophil staining. Bar: 50 μm. **b** Quantitation of macrophage-positive cells (*n* = 4). **c** Quantitation of neutrophil-positive cells. **d** Relative change of *Il-1b* transcript levels in kidney tissues (*n* = 3). **e** Relative change of *Tnf-α* transcript levels in kidney tissues (*n* = 3). The mRNA level of *Il-1b* and *Tnf-α* was normalized to the mRNA level of Gapdh in the same sample to determine the ratios, and the ratios of sham-operated WT were arbitrarily set as 1. Each symbol (circle and diamond) represents an individual mouse. Error bars: SEM. **p < 0.01; ***p < 0.001

Inflammation contributes critically to the initiation and extension of AKI^28^. We therefore evaluated renal inflammation by assessing renal infiltration of macrophages and neutrophils. As shown in Fig. 5a–c, renal IR-induced macrophage and neutrophil infiltration into kidneys in both *Bnip3*-KO and WT mice. Quantification analysis showed that ischemic *Bnip3*-KO mice had more macrophages (Fig. 5b) and neutrophils (Fig. 5c) infiltrating the kidney than ischemic WT mice. Moreover, quantitative real-time polymerase chain reaction (qRT-PCR) analysis of the expression of proinflammatory cytokines interleukin-1β (IL-1b) and tumor necrosis factor-α (TNF-α) in kidney tissues demonstrated that renal IR induced significantly higher expression of these cytokines in *Bnip3*-KO mice than in WT mice (Fig. 5d, e). These results support that *Bnip3*-KO mice have much stronger interstitial inflammation than WT mice after renal IR.

### *Bnip3* deficiency accumulates damaged mitochondria and increases ROS production following renal IR

**Fig. 6:**
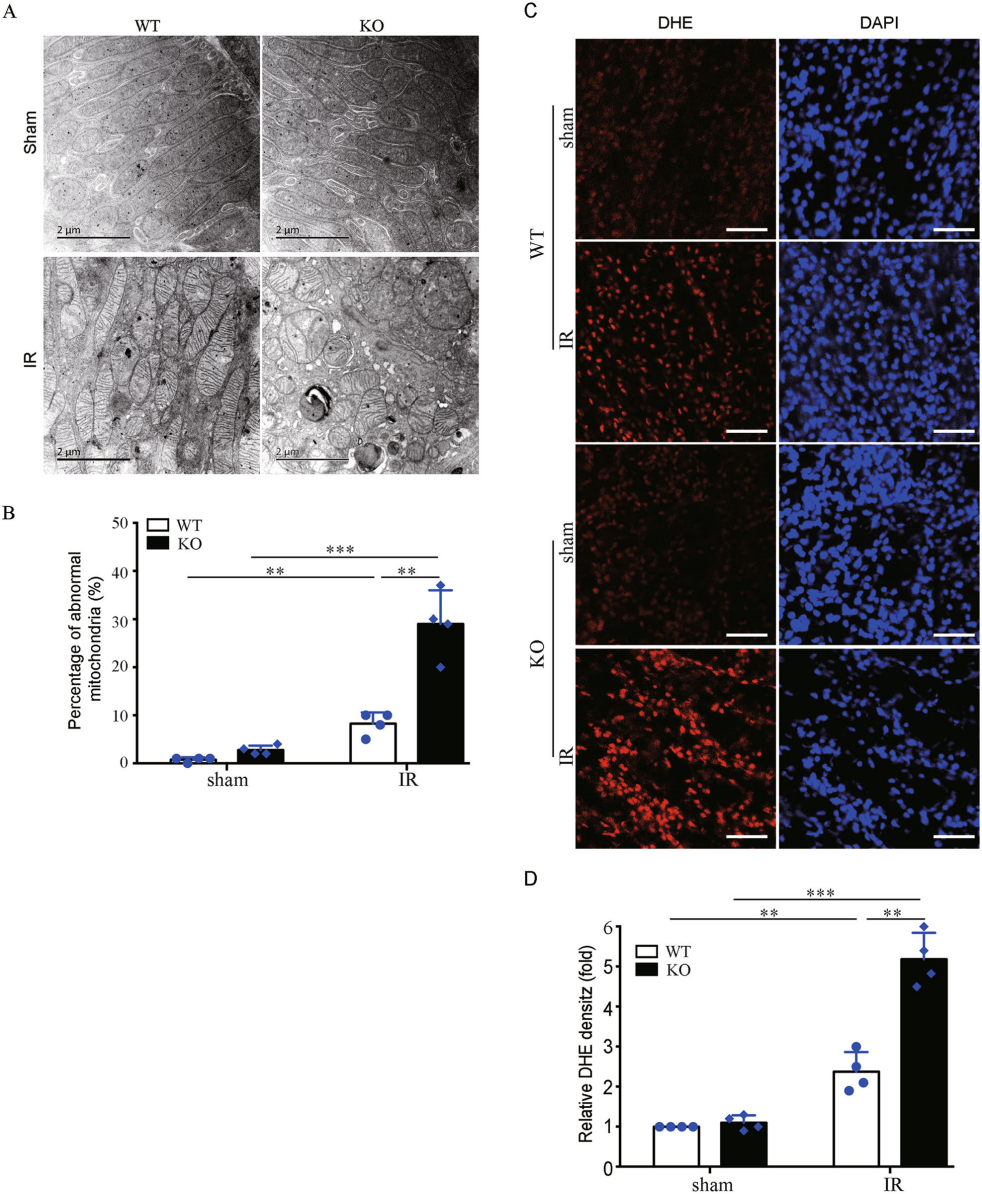
*Bnip3* deficiency accumulates damaged mitochondria and ROS production following renal IR. *Bnip3*-KO and WT mice were subjected to renal IR or sham operation (sham). Renal cortex was fixed and processed for TEM analysis and DHE staining. **a** Representative TEM images of mitochondrial morphology in proximal tubular cells. **b** Percentage of abnormal mitochondria (swollen with evidence of severely disrupted cristae over all mitochondria) (*n* = 4). **c** Representative images of DHE staining. DHE nuclear staining indicates the presence of reactive oxygen species (ROS) (*n* = 4). Scale bar: 100 μm. **d** Quantification of DHE fluorescence intensity. Each symbol (circle and diamond) represents an individual mouse. Error bars: SEM. **p < 0.01; ***p < 0.001

We examined the effect of *Bnip3* deficiency on mitochondrial integrity in RPTCs by transmission electron microscopy (TEM) and morphometric analysis as previously described^18,29^. Under controlled condition, *Bnip3*-KO and WT mice had tubular mitochondria in RPTCs (Fig. 6a). After renal IR, WT mice had an increase of small and round mitochondria, indicating mitochondrial fragmentation (Fig. 6a). Notably, loss of BNIP3 showed profound effects on mitochondrial structure, including the increase in mitochondrial fragmentation and swelling, vacuoles in the mitochondrial matrix, and loss of cristae. Quantification analysis of abnormal mitochondria that were swollen with evidence of severely disrupted cristae revealed that renal IR *resulted in* significantly more damaged mitochondria in RPTCs in *Bnip3*-KO mice than in WT mice (Fig. 6a, b). Collectively, these findings suggest that loss of *Bnip3* accumulates damaged mitochondria in renal tubular cells following renal IR. Mitochondrial damage and dysfunction are associated with increased ROS production. Thus, we determined ROS levels within kidney tissues by dihydroethidium (DHE) staining. Under the condition of sham operation, both WT and KO mice had similarly low intensity of DHE signals in RPTCs (Fig. 6c). However, KO and WT kidneys showed a dramatic increase in the DHE signals in RPTCs after renal IR (Fig. 6c), and quantification analysis demonstrated that the KO mice had significantly stronger DHE signals than WT mice (Fig. 6d). These results of DHE staining are in line with the findings of more accumulation of damaged mitochondrial in RPTCs of *Bnip3*-KO mice.

### *Bnip3* deficiency reduces renal IR-induced mitophagy in vivo

**Fig. 7:**
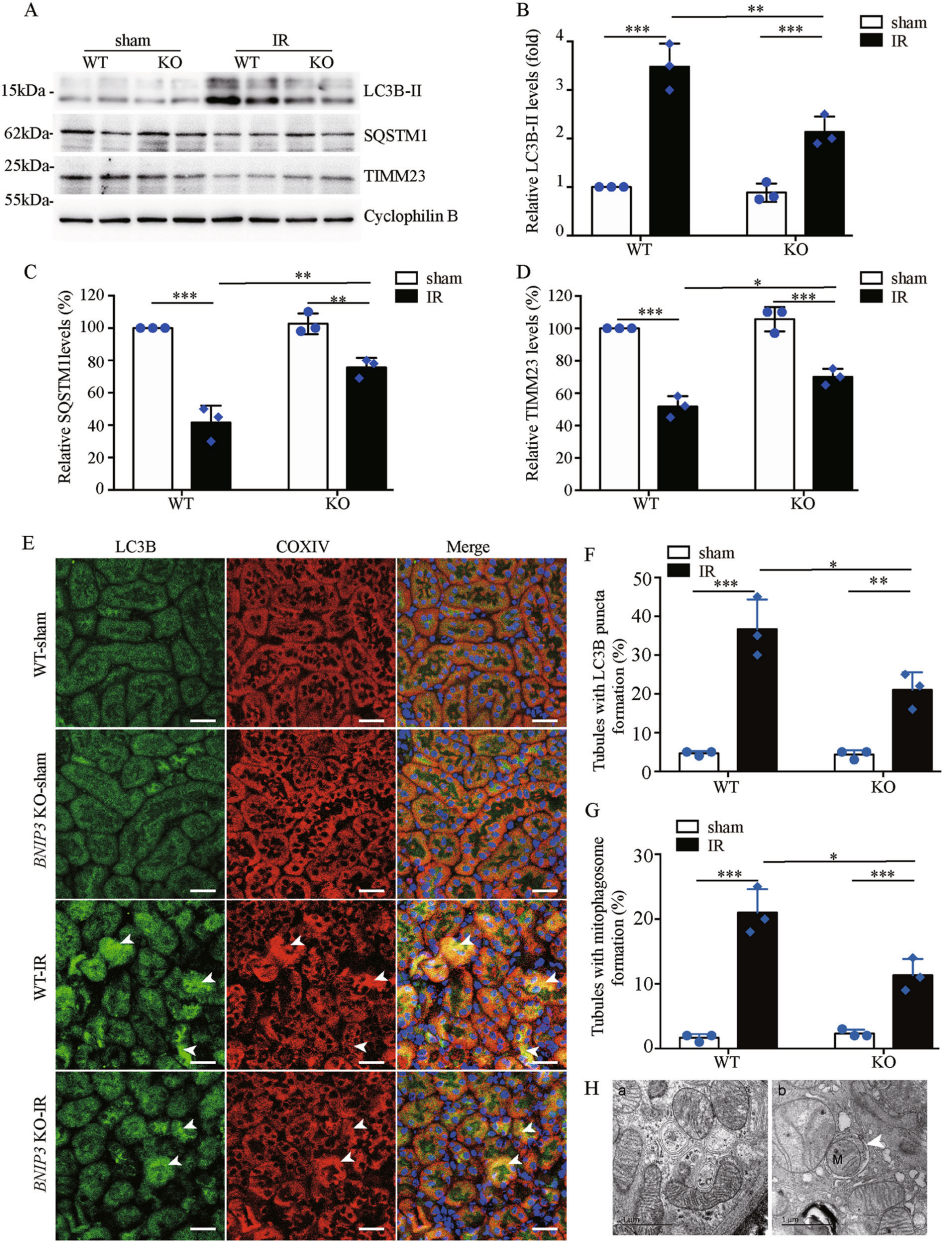
*Bnip3* deficiency suppresses renal IR-induced mitophagy. *Bnip3*-KO and WT mice were subjected to renal IR or sham operation (sham). Renal cortex was collected for immunoblot analyses of LC3B-I/II, SQSTM1, TIMM23, and cyclophilin B (loading control), immunofluorescence of LC3B and COXIV, and for transmission electronic microscopy (TEM) analysis. **a** Representative blots. **b**–**d** Densitometry of LC3B-II (**b**), SQSTM1 (**c**), and TIMM23 (**d**). For densitometry, the target proteins signals were normalized to the cyclophilin B signal of the same samples to determine the ratios, and the ratios of sham-operated WT were arbitrarily set as 1 or 100%. **e** Representative images of immunofluorescence staining of LC3B (green) and COXIV (red) and DAPI (blue). **f** Percentage of renal tubules with LC3B puncta. **g** Percentage of renal tubules with mitophagosome formation (LC3B puncta colocalizes with mitochondria) in renal tubules. Bar: 50 μm. **h** TEM images of mitophagosome (pointed by arrowhead in the right panel) in renal proximal tubule cells after IR. Each symbol (circle and diamond) represents an individual mouse. Error bars: SEM, *n* = 3. *P < 0.05; **p < 0.01; ***p < 0.001

The marked accumulation of damaged mitochondria in RPTCs of ischemia-operated *Bnip3*-KO mice suggests possibly defective mitochondrial elimination. Therefore, we evaluated the effect of loss of *Bnip3* on mitophagy in kidneys. Immunoblotting analysis showed an increase of LC3B-II and a concurrent decrease of SQSTM1 in WT kidney tissues after renal IR (Fig. 7a–c), and their changes were accompanied by a remarkable reduction of mitochondrial protein TIMM23 (Fig. 7a–d), suggesting mitophagy and mitochondrial clearance. Notably, the renal IR-induced changes in LC3B-II, SQSTM1, and TIMM23 in WT kidneys were partially but significantly reversed in ischemic *Bnip3*-KO kidney (Fig. 7a–d), suggesting an inhibitory effect of loss of BNIP3 on mitophagy. We further evaluated autophagosome and mitophagosome formation by immunofluorescence staining in kidney tissues using primary antibody against LC3B and mitochondrial protein cytochrome *c* oxidase subunit 4I1 (COX4I1/COXIV), respectively, and corresponding fluorescently labeled secondary antibodies. As shown in Fig. 7e, LC3B puncta were rarely observed in kidney tissues of sham-operated mice regardless of *Bnip3* status, but were dramatically increased in kidney tissue of ischemic *Bnip3*-KO and WT mice. Of note, partial LC3B-labeled autophagosomes colocalized with the mitochondria, indicating the formation of mitophagosomes (Fig. 7e). Quantification analysis demonstrated that there was significantly less renal tubules with autophagosome and mitophagosome formation in *Bnip3*-KO mice compared to WT mice after renal IR (Fig. 7e–g), suggesting a reduction of mitophagy under the condition of *Bnip3* deficiency. Further examination by using TEM confirmed autophagosome and mitophagosome formation in tubular cells after renal IR (Fig. 7h), which were rarely observed in kidney tissues from sham-operated mice. Taken together, these findings provide in vivo evidence that BNIP3 has a role in mitophagy regulation in renal tubular cells in ischemic AKI.

## Discussion

The pathophysiological role of BNIP3 in AKI remains unknown. In the present study, we have provided substantial evidence supporting a renoprotective role of BNIP3 in renal tubular cells via regulating mitophagy. We demonstrates that BNIP3 is upregulated in RPTCs in both in vitro and in vivo models of ischemic AKI, and loss of BNIP3 aggravates ischemic injury. We provide further evidence that loss of BNIP3 reduces mitophagy, resulting in the accumulation of damaged mitochondria and ROS, increases of cell death, and enhancement inflammatory response in kidneys following renal IR. Thus, these findings indicate that enhancement of mitophagy may represent a promising novel therapeutic approach for AKI.

Upregulation of BNIP3 in RPTCs has been implicated in an experimental rat model of ischemic AKI^20^. However, the pathophysiological role of BNIP3 in AKI pathogenesis remains largely unknown. BNIP3 was initially identified as a pro-death protein^22^, and its pro-death function has been associated with the pathogenicity of cardiac diseases^30–33^. For instance, in animal models of cardiac ischemia-induced myocardial infarction, BNIP3 induction in ventricular myocytes occurred during early cardiac ischemia and persisted throughout reperfusion in an accompany with mitochondrial dysfunction and cardiomyocyte death^32^. More importantly, inhibition of BNIP3 activity by gene deletion or expression of dominant-negative form of BNIP3 dramatically reduced myocardial apoptosis and myocardial injury after cardiac IR^30,34^, suggesting a pathogenic role of BNIP3 induction in cardiac infarction. On the other hand, emerging evidence also supports a cytoprotective role of BNIP3 in some disease conditions^24–27^. Our present study provides substantial evidence supporting a renoprotective role of BNIP3 in ischemic AKI, as evidenced by much more severe kidney structural and functional damage (Fig. 3d–h), more tubular cell death (Fig. 4), and stronger inflammation (Fig. 5) in the kidney of *Bnip3*-KO mice compared to their WT littermates after renal IR. The discrepancy concerning BNIP3 function in different tissues may suggest cell-type-specific functions of BNIP3. However, the underlying determinants of the pro-survival or pro-death functions of BNIP3 in different conditions remains poorly understood.

BNIP3 has recently been recognized as a regulator of mitophagy^24,25^, but its pro-mitophagy function in kidney remains largely unknown. In present study, several lines of evidence supported an occurrence of BNIP3-mediated mitophagy in renal tubular cells in ischemic AKI, which includes: (1) upregulation of BNIP3 was associated with an increase of autophagy flux and mitochondrial proteins degradation, as well as mitophagosome formation in renal tubular cells following renal IR (Figs. 2 and 7); (2) all of these above changes were partially reversed by loss of BNIP3 (Figs. 2 and 7); (3) loss of BNIP3 led to the accumulation of damaged mitochondria following renal IR (Fig. 6). Of note, recent studies from us and others have demonstrated an induction of PINK1-PARK2 pathway of mitophagy in RPTCs during AKI^17–19^. Collectively, these findings strongly support an involvement of mitophagy in AKI pathogenesis, and, moreover, mitophagy in AKI may involve multiple pathways. Recent studies also provide evidence that there exists cross-talks between different mitophagy pathways. For instance, in cardiac myocytes, PARK2 translocation to mitochondria was demonstrated to be essential for BNIP3-mediated mitophagy^35^. More recently, Zhang et al.^24^ provided evidence that BNIP3 binding to PINK1 suppressed PINK1 proteolytic cleavage and therefore increased the accumulation of intact PINK1 on the outer membrane of mitochondria, leading to increased PARK2 recruitment to the mitochondria and consequent mitophagy induction. Thus, it will be interesting to investigate whether BNIP3 and PINK1/PARKIN pathways of mitophagy act coordinately in regulating renal tubular cell mitophagy in AKI.

Mitophagy is a critical component of the mitochondrial quality control, which is critical for the maintenance of a healthy mitochondrial population and thereby cell viability. Under conditions when damaged mitochondria overwhelm the capacity of mitophagy, and/or mitophagy is impaired, damaged mitochondria accumulate, which may result in excessive ROS production, activation of cell death, and release of mitochondrial DAMPs. In consistent with previous reports, we verified that renal IR induced mitochondrial damage and increased ROS production in renal tubular cells, and, more importantly, these changes were aggravated within kidneys of *Bnip3*-KO mice in the same experiment (Fig. 6). It is conceivable that failing of BNIP3-mediated mitophagy expands renal IR-induced mitochondrial damage, which leads to further damage to the mitochondria and ultimately irreversible cell injury and cell death. Moreover, ROS, mitochondrial DAMPs, and various factors or contents released from necrotic tubular cells may stimulate robust inflammation to contribute tissue damage. In line with this notion, renal IR induced a stronger inflammatory response in the kidney of *BNIP3*-KO mice than in WT mice, as evidenced by significantly more macrophage and neutrophil infiltration into kidneys and higher expression of proinflammatory cytokines in these KO mice compared to WT mice (Fig. 5).

In summary, this study has provided substantial evidence for the activation of BNIP3-mediated mitophagy in RPTCs in ischemic AKI. We provided further evidence that BNIP3-mediated mitophagy has important role in mitochondrial quality control, tubular cell survival, and renal function during AKI. Notably, previous reports suggest that induction of PINK1/PARKIN pathways of mitophagy protects against AKI. As such, enhancing mitophagy may offer a novel therapeutic strategy for AKI.

## Material and methods

### Cells, plasmids, and transfection

BUMPTs were originally from Dr. Wilfred Lieberthal (Boston University School of Medicine) and maintained as described previously^36^. *Bnip3*-KD cells were generated by infection of BUMPT cells with *Bnip3*-shRNA-encoding lentivirus. Briefly, shRNAs specifically targeting mouse *Bnip3* were synthesized by GenePharma (China), and then inserted into lentivirus vector PLKO.1 (Addgene plasmid #8453). After viral packaging, the viruses encoding *Bnip3* shRNA were harvested to infect BUMPT cells. Infected cells were selected with 2.5 mg/ml puromycin (Sigma-Aldrich) to generate stable cell line with *Bnip3* KD. The KD efficiency was determined by qRT-PCR and immunoblotting analysis. The shRNA target sequences of *Bnip3* were as follows: forward, 5′-CAGCCTCCGTCTCTATTTA-3′; reverse, 5′-TACCAACAGAGCTGAAATA-3′. The GFP-LC3B plasmid was obtained from Addgene (#11546). Transfections of BUMPT cells with plasmid DNAs were performed by using Lipofectamine 3000 reagents (L3000008; Thermo Fisher Scientific) under the manufacturer’s instruction.

### Antibodies and reagents

Anti-SQSTM1 (5114), anti-COXIV (11967), and anti-activated caspase-3 (9664) were from Cell Signaling Technologies; anti-LC3B (NB100-2220) were from Novus Biologicals; anti-TIMM23 (sc-514463) and anti-TOMM20 (sc-11415) were purchased from Santa Cruz Biotechnology; anti-KIM1/HAVCR1 (hepatitis A virus cellular receptor 1) from R&D Systems; anti-cyclophilin B, anti-macrophage (RM0029-11H3), anti-neutrophil (NIMP-R14), and anti-BNIP3 (ab109362, ab10433) were obtained from Abcam; anti-β-actin (A5316) was from Sigma-Aldrich. All secondary antibodies, DHE (D11347) and collagen I (A1048301), were purchased from Thermo Fisher Scientific, and TUNEL assay kit (12156792910) was obtained from Roche Life Science.

### Oxygen-glucose deprivation-reperfusion

OGD-R in BUMPT cells was performed as previously described with modification^37^. Briefly, BUMPT cells were seeded in collagen I-coated plates at 24 h before treatment. Cells at an ~95% confluence were rinsed once with glucose-free Dulbecco’s modified Eagle’s media (Thermo Fisher Scientific, 11966), and then maintained in both O2 and glucose-free medium. Cells were immediately placed in a sealed incubator chamber (Billups-Rothenburg, MIC-101), and then loaded with N2 gas for 10 min at 25 L/min. After incubation at 37 °C for 2 h, cells were transferred back to normal culture medium and regular incubator with oxygen for reperfusion. Cells cultured in normal medium and regular cell incubator with 21% oxygen were used as control.

### Animals and renal IR

Animal experiments were conducted in accordance with a protocol approved by the Institutional Animal Care and Use Committee of the Second Xiangya Hospital of Central South University. C57BL/6 mice were purchased from SJA Laboratory Animal Corporation (Hunan, China). *Bnip3*-KO mice were previously described^24^. Mice were housed in a pathogen-free condition under cycles of 12:12-h light and dark with free access to food and water. Renal IR surgery in mice was performed as previously described^18^. Briefly, bilateral renal pedicles were exposed for clamping to induce 30 min of ischemia, and the clamps were then released for reperfusion for 48 h. Mice underwent the same operation without renal pedicle clamping were as sham control mice.

### Renal function and histopathology

Serum creatinine was evaluated to determine renal functions with a kit from BioAssay Systems (DICT-500) as previously described^18^. To determine tissue damage, paraffin-embedded kidney tissue sections with a thickness of 4 µm were stained with HE. Renal tubules with loss of brush border, tubular dilation and disruption, cast formation, and cell lysis were considered damaged. Tissue damage was evaluated by the percentage of damaged tubules: 0, no damage; 1, <25%; 2, 25–50%; 3, 50–75%; 4, >75%.

### Immunohistochemical analysis

Immunohistochemical staining was performed as described in our recent studies^18^. Briefly, paraffin-embedded kidney sections were sequentially underwent deparaffinization, hydration, and antigen retrieval by incubation with 0.1 M sodium citrate, pH 6.0 at 100 °C. After subsequent incubation in 3% H_2_O_2_, 5% normal donkey serum and 0.1% Triton X-100 to reduce non-specific binding, tissue sections were exposed to 1:100 anti-BNIP3 (Abcam, Ab10433), 1:200 anti-cleaved caspase-3 (5A1E) (Cell Signaling, 9664), 1:100 anti-macrophage (Abcam, RM0029-11H3) or 1:100 anti-neutrophil (Abcam, NIMP-R14) at 4 °C overnight followed by exposure to horseradish peroxidase (HRP)-conjugated secondary antibody for 1 h at room temperature. Signals of the antigen–antibody complexes were detected with a DAB Peroxidase Substrate Kit (Vector Laboratories) following the manufacturer’s introduction. Sections were then counterstained with DAPI (4′,6-diamidino-2-phenylindole) (Sigma-Aldrich, D9542). For quantification, 10–20 fields were randomly selected from each tissue section and the amounts of positive cells per mm^2^ was evaluated.

### TUNEL assay

TUNEL assays were performed to evaluate cell apoptosis in kidney tissue and BUMPT cells using an In Situ Cell Death Detection Kit (Roche Applied Science, Indianapolis, IN) as described in our recent studies^29,38^. For quantification, the number of TUNEL-positive tubular cells in kidney sections in at least 10 optical fields in each tissue section from three different kidneys per group was counted, and the amount of TUNEL-positive cells per mm^2^ was evaluated. For quantification of TUNEL-positive BUMPT cells, at least 10 optical fields with >200 cells from three different experiments were examined in each condition to estimate the apoptosis percentage.

### Determination of ROS

DHE staining of frozen kidney tissues were performed to elevated ROS levels as previously described^18^. Briefly, after harvest, kidney tissues were immediately placed in Tissue-Tek optimal cutting temperature compound (Sakura Finetek, 4583), followed by snap freezing in liquid nitrogen. Unfixed frozen kidney sections of 20 μm thickness were incubated in 10 μM DHE at 37 °C for 30 min followed by counterstaining with DAPI (Sigma-Aldrich, D9542). Samples were imaged with confocal microscopy (Leica, TCS SP5). For quantification, the fluorescent density in the nuclei of proximal tubular cells within 10 random optical field from three different kidneys per group was determined with the ImageJ software.

### Transmission electron microscopy

TEM analysis was performed as recently described^29^. Immediately after harvest, kidney tissues were sequentially fixed in paraformaldehyde and glutaraldehyde (Sigma-Aldrich, 340855), post fixation in osmium tetraoxide (Sigma-Aldrich, 201030), and dehydration in ethanol. Samples were then embedded in Epon (Sigma-Aldrich, 45345). Seventy-nanometer-thick tissue sections were stained with uranyl acetate (TED PELLA, 19481) and lead citrate (Sigma-Aldrich, 15326). Samples were imaged by using the TEM (Leica). For quantification of abnormal mitochondria that were swollen with severely disrupted cristae, at least 400 individual mitochondria from four different kidneys per group were counted. The percentage of abnormal mitochondrial over all mitochondria was analyzed to indicate the degree of mitochondrial damage.

### Quantitative real-time PCR

Total RNA from cells and kidney tissues was extracted with TRIzol reagent (Thermo Fisher Scientific, 15596026) according to the manufacturer’s instruction. Complementary DNA was synthesized using reverse transcription reagents (Thermo Fisher Scientific, N8080234). Quantitative real-time PCR was performed with the TB Green Premix Ex Taq II reagent (TaKaRa, RR820B) on LightCycler96 Real-Time PCR System (Roche Life Science). For quantification, the mRNA levels of target genes were normalized to the mRNA levels of *Gapdh*. Primers used were as follows: *Bnip3*—forward, 5′-GCTCCCAGACACCACAAGAT-3′ and reverse, 5′-TGAGAGTAGCTGTGCGCTTC-3′; *Tnf-α*—forward, 5′-CAGGCGGTGCCTATGTCTC-3′ and reverse, 5′-CGATCACCCCGAAGTTCAGTAG-3′; *Il-1b*—forward, 5′-GAAATGCCACCTTTTGACAGTG-3′ and reverse, 5′-CTGGATGCTCTCATCAGGACA-3′; *Gapdh*—forward, 5′-AGGTCGGTGTGAACGGATTTG-3′ and *Gapdh* reverse, 5′-GGGGTCGTTGATGGCAACA-3′.

### Immunofluorescent staining

Immunofluorescence staining in cultured cells was performed as previously described^24^. Briefly, cells were washed once with phosphate-buffered saline (PBS), followed by fixation with 4% paraformaldehyde and subsequent permeabilization with 0.1% Triton X-100. After being blocked with 5% bovine serum albumin, cells were probed with 1:100 anti-LC3B and 1:100 anti-COXIV, followed by exposure to Alexa-conjugated secondary antibodies. Samples were imaged using a confocal microscope (Leica, TCS SP5). For quantification, the number of LC3B puncta and LC3B puncta colocalizing with the mitochondria from at least 30 cells in three independent experiments were counted. For immunofluorescence staining in kidney tissues, parafilm-embedded tissue sections underwent deparaffinization, rehydration, and antigen retrieval by incubation with 1 mM EDTA (pH 8.0) at 95–100 °C for 1 h. After being subsequently incubated with 3% H_2_O_2_, 5% normal donkey serum, and 0.1% Triton X-100. Sections were exposed to primary antibodies at 4 °C overnight and detected with Alexa-conjugated secondary antibodies. Samples were imaged using a confocal microscope (Leica, TCS SP5). For quantification, the number of LC3B puncta and LC3B puncta colocalizing with mitochondrial signals from 10 to 20 random fields (×400) for each slide from three different kidneys was counted to indicate autophagosome and mitophagosome formation.

### Immunoblot analysis

Cells or kidney tissues were washed with PBS buffer and then lysed in sodium dodecyl sulfate (SDS) sample buffer (63 mM Tris-HCl, 10% glycerol, and 2% SDS) containing protease inhibitor cocktail (Sigma-Aldrich). Protein concentration was determined by the BCA method with reagents from Thermo Scientific. Equal amount of proteins from different groups were separated by electrophoresis. After transferring onto polyvinylidene difluoride membrane, the membrane was sequentially incubated with blocking buffer (5% skim milk), a primary antibody and a corresponding HRP-linked secondary antibody. The target protein was visualized using chemiluminescent substrate (Thermo Scientific). The protein band intensity was quantified by the ImageJ software (NIH).

### Statistical analysis

Statistical analysis was conducted with the Prism software (GraphPad). Statistical differences in multiple groups were determined by multiple comparisons with analysis of variance, followed by Tukey’s post tests. Differences between two groups were determined by two-tailed unpaired or paired Student’s *t* test. Quantitative data were expressed as means ± SEM.
